# Feasibility of Ultrasound-Assisted Extraction for Accelerated Cold Brew Coffee Processing: Characterization and Comparison With Conventional Brewing Methods

**DOI:** 10.3389/fnut.2022.849811

**Published:** 2022-03-18

**Authors:** Xingchen Zhai, Mengnan Yang, Jing Zhang, Lulu Zhang, Yarong Tian, Chaonan Li, Lina Bao, Chao Ma, A. M. Abd El-Aty

**Affiliations:** ^1^Beijing Key Laboratory of Forest Food Processing and Safety, College of Biological Science and Technology, Beijing Forestry University, Beijing, China; ^2^Department of Pharmacology, Faculty of Veterinary Medicine, Cairo University, Giza, Egypt; ^3^Department of Medical Pharmacology, Faculty of Medicine, Atatürk University, Erzurum, Turkey

**Keywords:** coffee, cold brew, hot brew, ultrasound-assisted extraction, volatile compounds

## Abstract

A long extraction time for traditional cold coffee brewing considerably reduces the production efficiency of this type of beverage. Herein, a new ultrasound-assisted cold brewing (UAC) method was established. The feasibility of UAC was assessed by comparison with main physicochemical characteristics, non-volatile and volatile compounds in coffee extracts produced by hot brewing and conventional static cold brewing methods. Compared to the static cold brews, the levels of total dissolved solids, total lipids, proteins, and titrated acids of UAC coffee extracts increased by 6–26%, 10–21%, 26–31%, and 12–15%, respectively. Caffeine, chlorogenic acid, and trigonelline concentrations were also determined by HPLC. Based on the volatile profiles obtained by HS-SPME-GC/MS, the aroma compounds in UAC was significantly different (*p* < 0.05) from hot brews but similar to static cold ones, suggesting that ultrasonication compensated for the time of the static cold brewing, thereby considerably shortening the extraction time (1 h vs. 12 h). This work demonstrated that the combination of ultrasound-assisted with cold brew could produce coffee with good flavor and increase the extraction efficiency, which may provide an option for the acceleration of the cold brew coffee process.

## Introduction

Coffee is the most widely consumed beverage worldwide and one of the most commercial food products. From an engineering point of view, coffee brewing is a solid–liquid extraction, where the roasted and ground coffee is in intimate contact with water. Depending on the extraction technique, water acts as a solvent to extract soluble and non-soluble compounds. These compounds eventually appear in the form of dissolved or suspended solids in the extract, which substantially impact the sensory properties of coffee (1). Traditionally, coffee has been prepared with hot water (near the boiling point) within just a few minutes (2). The high temperature is the driving force for extraction. With the increasing demands for coffee with unique sensory characteristics, cold brew coffee has emerged and spread rapidly, which is prepared with water at 20–25°C or lower temperatures and requires a more extended period than the conventional hot brew methods, varying from 8 to 24 h (2, 3). The unique extraction conditions make cold brewing coffee have an utterly different flavor than hot brewing coffee, manifested as intense sweetness, chocolate, floral and fruity aroma, with moderate bitterness and acidity (4). According to market analysis, the global market size for cold brew coffee was valued at USD 339.7 million in 2018 and is expected to reach USD 1.63 billion by 2025 (5).

Significant differences were found in the chemical, physical parameters, and sensory profiles (such as bitter, sweet, astringency) between cold brew and cold drip coffee extractions (6). Rao et al. (7) indicated that cold brew coffees showed decreased acidity, fewer total dissolved solids (TDS), and a lower concentration of browned compounds than hot brewing extractions. According to Fuller and Rao (8), caffeine and chlorogenic acid (CGA) concentrations reached an equilibrium between 6 and 7 h in cold brew samples based on first-order kinetics. Compared to hot brew coffee, substantially higher caffeine concentrations were found in cold counterparts, while there were no significant differences in CGA concentrations. Despite the physicochemical properties of classic cold brew coffee research, there are a few studies on alternative methods to reduce the long cold extraction time. Recently, Morgan Caudill (9) accelerated cold brew extraction through microwave heat treatment, but the temperature still needs to reach up to 80°C. Ultrasound-assisted extraction is a green and economical technology with high efficiency for food and natural products, and it acts with combined mechanisms between fragmentation, erosion, capillarity, detexturation, and sonoporation (10). Ultrasound has been reported to increase the yield of flavonoids extraction from sea buckthorn (11), saponin from ginseng (12), and triglycerides from coffee (13). Probably, ultrasound-assisted is an alternative method to improve the cold brewing efficiency and to create faster commercialization of cold brewing.

Therefore, the present study aimed to explore the feasibility of ultrasound-assisted cold brew (UAC) as an alternative extraction method to shorten the long extraction time required by the traditional cold brewing methods.

## Materials and Methods

### Materials and Reagents

Arabica coffee (originating from Brazil) from Basic ElementsCatering Services Co., LTD (Gu'an, China) were was used in all the trials. Caffeine, CGA, and trigonelline were obtained from Chengdu Effa Biological Technology Co., Ltd (Chengdu, China). Folin–Ciocalteu was purchased from Coolaber Technology Co., Ltd (Beijing, China). Other chemicals, such as gallic acid, sodium carbonate, *n*-hexane, and sodium chloride, were of analytical grade and secured from Sinopharm Chemical Reagent Co., Ltd (Shanghai, China).

### Coffee Extracts Preparation

Roasted coffee beans were ground until they passed through a 20-mesh sieve. All samples were prepared using the same commercial brand of mineral water and the same coffee to water ratio (1:18).

For hot brewing, hot boiled (HB) coffee was prepared with coffee (16.5 g) and hot water (313.5 g, 95°C) was mixed for 5 min. Three-stage water (92°C) injection was performed for pour-over (PO) coffee. Firstly, 30 g water was added to coffee to stew and steam for 40 s. Then water was slowly added to 150 g. Finally, the remained hot water was injected to complete the extraction for a total time of about 3 min. All filtered samples from different methods were used for further analysis.

Cold brew coffee was prepared by immersion methods performed under static (4 or 10°C) or ultrasonic conditions. Conventional cold brews were produced by coffee immersed in water at 4 or 10°C for 12 h (short for 4 and 10B). UAC coffee was prepared by probe-ultrasound equipment with 200 W for 60 min (Biosafer 3D, Saifei China Technology Co., LTD) at room temperature. When extraction ended, the beverage was filtered through a paper coffee filter for further analysis.

### Physicochemical Analysis

#### Extraction Yield, Color Values, pH, and TDS

The following equation measured the extraction yield (EY): EY(%) = (W_2_ − W_1_)/W_0_ × 100%, where W_2_ defines the total mass of extract obtained in the evaporating dish, W_1_ as the empty evaporating dish mass, and W_0_ as the initial coffee mass used in the extraction.

A Chroma meter (SR60, Sanenshi, Shenzhen, China), pH meter (LE438, METTLER TOLEDO, Zurich, Switzerland), and refractometer (LH-Q20, Luheng, Hangzhou, China) were used to measure color (*L, a, b* values), pH, and total dissolved solids (TDS) of different coffee extracts, respectively.

#### Extraction Rate of Total Phenols (TPC), Lipids (TL), Proteins (Tpro), and Titratable Acidity (TA)

Total phenols was measured using the Folin-Ciocalteu method described by Cordoba et al. (2) with minor modifications. A 0.1 mL coffee extraction sample, 6 mL distilled water, 0.5 mL Folin-Ciocalteu reagent, and 1.5 mL 20% Na_2_CO_3_ solution were added, followed by distilled water to make 10 mL. All mixed samples were incubated at room temperature for 2 h before measuring the absorbance at 765 nm. The results were expressed as total phenolic content in micrograms of gallic acid equivalents per millilietr of solution. After that, TPC was calculated by TPC(%) = c × V × N/W × 100%, where c defined as total phenolic content (μg/mL); V is the volume of coffee extracts (mL); N as diluted multiples; and W is the coffee mass corresponding to the coffee extraction (μg).

Soxhlet extraction (SE) was used to evaluate TL as recommended by the Association of Official Analytical Chemists (AOAC). SE was conducted for 6–8 h with 10 mL of coffee extraction sample and petroleum ether in a water bath, and then the suspension was filtered with filter paper. Petroleum ether was evaporated in the fume hood. The lipids extracted were placed into a vacuum drying oven until they reached a constant mass. Afterward, TL (%) in coffee was obtained by gravimetric analysis, which was expressed as lipids amount extracted from coffee per unit mass.

Total protein extraction rate was determined by the Kjeldahl method. A 10 mL coffee sample was put in the digestive tube, then 4 g potassium sulfate, 0.25 g copper sulfate pentahydrate, and 10 mL concentrated sulfuric acid were added consecutively. Following total digestion in a furnace in a fume hood for 1–2 h, a fully automated Kjeldahl apparatus (KDY-9830, Tongrunyuan Co., Beijing, China) was used to measure the protein content, and Tpro (%) was expressed as protein amount extracted from coffee per unit mass.

For TA, 20 mL of coffee extraction was titrated with 0.1 mol/L NaOH solution at a pH of 6.5, and the TA (%) was expressed as CGA amount extracted from coffee per unit mass.

#### Caffeine, CGA, and Trigonelline Measurement

Caffeine, CGA, and trigonelline were measured by HPLC (LC-20A, Shimadzu Co., Kyoto, Japan) fitted with a Diamosil C18 column (Dikma, Beijing, China, 250 mm × 4.6 mm) run at 30°C with a mobile phase flow rate of 1.0 mL/min and an injection volume of 10 μL. For caffeine, separation was carried out with an isocratic gradient of a mixture of 90% mobile phase A and 10% mobile phase B (A: 0.1% aqueous phosphoric acid; B: acetonitrile). CGA was determined using the same mobile phase as caffeine, while the isocratic gradient was set to a mixture of 75% mobile phase A and 25% mobile phase B. As for trigonelline, elution was performed using an isocratic gradient of a mixture of 80% mobile phase A and 20% mobile phase B (A: 0.05%SDS-0.1% aqueous acetic acid; B: 0.1% acetic acid in methanol). Caffeine, CGA, and trigonelline were detected using a diode array detector at 272, 327, and 265 nm, respectively. Five concentrations for each compound were used to prepare the calibration curve, and regression equations were fitted by plotting of area of the standard solutions against concentrations.

### Volatile Compound Analysis

The volatile compounds from hot and cold brew coffee extracts were obtained by headspace–solid-phase microextraction (HS-SPME) and analyzed by gas chromatography coupled with mass spectrometry (GC/MS), according to the method reported by Moreno et al. (14) with minor modifications. Each extraction sample (2 mL) was equilibrated with 2 g sodium chloride for 30 min in a 25-mL sealed vial at 60°C using a magnetic stirrer with a speed of 260 rpm. The volatile compounds released from the headspace of each sample were collected using 50 μm/DVB/CAR/PDMS long fiber (SAAB-57348U, Anpu Experimental Technology Co., Shanghai, China) and directly injected into a gas chromatography–mass spectrometry (QP2010 Ultra, Shimazu, Tokyo, Japan). A RTX-5MS capillary column (Restek, Bellefonte, PA, USA, 30 m × 0.25 mm i.d., 0.25 μm) was used. The column oven was programmed from 40°C (after 5 min) to 120°C at 5°C/min, then to 280°C at 10°C /min, and maintained at the final temperature for 5 min. The injector and ion source temperatures were maintained at 280 and 230°C, respectively. The carrier gas was 1 mL/min of He, with a split injection of 40:1. Qualitative elucidation of the volatile compounds was performed by comparing their mass spectra with those of NIST14 or NIST14s mass spectra library. Quantification was conducted by the internal standard method using 4-methyl-2-amyl alcohol as the internal standard.

To assess the influence of the volatile compounds of coffee extraction, the odor activity values (OAVs) were calculated by the following equation: OAV_i_ = C_i_/OT_i_, where C_i_ referred to the concentration of a particular aroma in coffee extraction (μg/L), and OT_i_ referred to the aroma threshold in water from literature (μg/L) (15). Only compounds with an OAV > 1 contributed to the coffee aroma.

### Orthogonal Experimental Design

An L_9_ (3^3^) OED was used to obtain the optimal conditions for the production of UAC coffee with three operational parameters, including coffee to water ratio (1:12, 1:15, and 1:18), extraction time (20 min, 30 min, and 60 min), and ultrasonic power (100 W, 150 W, and 200 W) in three levels, respectively. The EY (%) of coffee was chosen as the observed index. The parameters, their ranges, and levels are listed in Table 1. Also, the quality parameters of the coffee obtained under optimized conditions were evaluated according to the methods displayed in Section Physicochemical Analysis.

**Table 1:** Factors and levels of OED L_9_ (3^3^) design.

**Independent variable**	**Range and levels**		
	**1**	**2**	**3**
Coffee to water ratio	1:12	1:15	1:18
Extraction time (min)	20	30	60
Ultrasonic power (W)	100	150	200

### Statistical Analyses

All measurements were carried out three times. One-way analysis of variance (ANOVA), followed by Ducan's test at a 5% significance level, was applied using SPSS Statistics 22.0. The result figures were drawn based on Microsoft Excel 2010, Origin Pro 2019, and SIMCA 14.1.

## Results and Discussion

### Comparison of Extraction Effects of Hot- and Cold-Brewing

Coffee comprises carbohydrates, lipids, proteins, melanoidins, organic acids, CGA, and nitrogen-containing compounds, such as caffeine and trigonelline (16). The chemicals in roasted coffee are extracted at different rates due to water solubility variations. EY is the ratio of the mass of extracted coffee solubles to the mass of the coffee grains used, which determines the coffee's flavor. The EYs of hot- and cold-brewing were evaluated using the same ratio of coffee/water. As shown in Supplementary Table 1, hot-brewing exhibited higher EYs than cold-brewing. The extraction temperature for hot-brewing was near 100°C, which is a high-energy system leading to a faster rate of diffusion of coffee grain in water and the release of components in the process of solid–liquid mass transfer (17). Regarding cold-brewings, the EY of UAC was higher than 4 and 10 CB, with significantly shorter extraction time, which may be because the acoustic cavitation that occurred throughout ultrasonication can accelerate the release, diffusion, and dissolution of the active components in coffee. Ahmed et al. (18) also proposed that the combination of ultrasonication and agitation enhanced various phytochemicals in cold-brew coffee, such as color values, TDS, antioxidant activities, and most organic acids. According to Brewing Control Charts of the Specialty Coffee Association (SCA), the EY should be 18–22%, with 0.79–1.38% TDS. With the emergence of more and more new brewing methods, the applicability of this classic chart developed in the 1950s is rapidly decreasing (19). Therefore, the ranges of EY and TDS for the present study were acceptable.

Soluble sugars are the main components of dissolved solids in coffee, mainly influencing the sweetness and viscosity (20). Compared with other methods, UAC exhibited the highest TDS (Supplementary Table 1). More water can move into the cells because the tissue and cell wall disruption by sonoporation during ultrasonication leads to increased permeability of cell membranes and more soluble solids passing through the cell membrane (18), which compensates for the time effect cold-brew at 4 and 10°C. The higher TDS may make UAC coffee sweeter, also with a relatively high pH. In this study, cold brew coffees displayed higher pH values (less acidic) than hot counterparts. Several pieces of research have reported that the pH, together with TA, in hot coffee extracts was higher than that measured in cold coffee extracts, indicating hot-brewing was able to extract more acids and additional acidic compounds (2), as the solubility of organic acids increased with temperature (21). The pH measurement mainly quantifies the concentration of hydrogen ions in an aqueous solution. At the same time, TA evaluates all acidic protons, including undissociated protons that a strong base can neutralize. Although many researchers want to establish correlations between pH, TA, and perceived acidity, no agreement has been reached as yet (19). In this work, UAC extractions presented the highest pH value and a relatively high TA, which might make the UAC coffee less acidic than the others. However, this needs further sensory evaluation to be verified.

**Figure 1:**
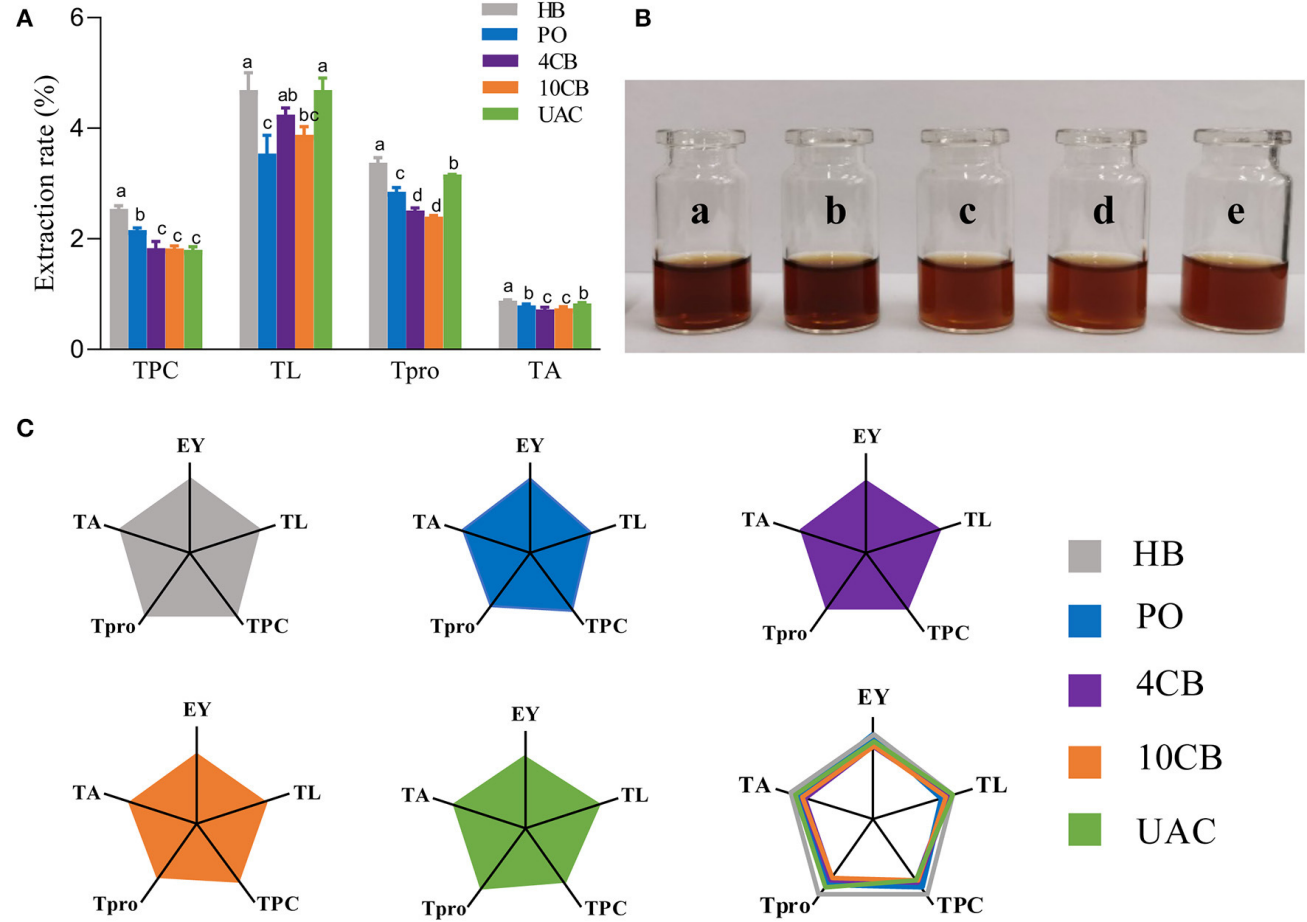
**(A)** The effects of different brewing methods on TPC, TO, Tpro, and TA. Different letters indicate statistically significant differences (*p* < 0.05) among treatments. **(B)** Actual images of a) hot boiled, b) pour-over, c) 4°C cold brew, d) 10°C cold brew, and e) ultrasound-assisted cold brewed coffee samples. **(C)** Spider plots of main physicochemical characteristics yielded by hot and cold brewing coffee methods. EY, Extraction yield; TL, total lipid extraction rate; TPC, total phenolic extraction rate; Tpro, total protein extraction rate; TA, total acid extraction rate. HB, Hot boiled, PO, Pour-over, 4 CB, 4°C cold-brew, 10 CB, 10°C cold brew, UAC, Ultrasound-assisted cold brew.

Generally, raw coffee beans are green in color. As the temperature rises during roasting, the Maillard reaction and Strecker degradation give the beans a new color and aroma. Herein, the values of a^*^ and b^*^ for all samples were close to zero, so the red–green, and yellow–blue differences could be ignored. While hot-brewing coffee had lower L^*^ and a^*^ values, indicating darker color in hot-brewing extracts, consistent with actual images (Figure 1B), cold-brewing coffees exhibited hazelnut color with a hint of brown, and the color of UAC counterpart was the brightest, owing to the highest L^*^ value observed. Also, cold coffee brews showed significant differences compared with hot ones with regard to TPC, TL, Tpro, and TA (Figure 1A). The TPC values were comparable in the cold brews, significantly lower than HB and PO coffees. Several phenolic substances in coffee, such as CGA, will be accelerated to release by high temperatures. This is also the reason for the highest TPC and TA of HB coffee. Since coffee's sour and astringency flavor is mainly provided by phenols and their degradation products (22), low TPC makes UAC coffee slightly sweet and tasty. Some shear forces are generated within the liquid (water) for total lipids and proteins extraction by UAC. The vicinity of solid materials (coffee) and the collapse of a cavitation bubble close to the cell could cause its rupture (23), making UAC coffee exhibit a relatively high TL and Tpro. Lipids in coffee brews can form emulsions that retain aromatic compounds to strengthen the aroma and provide coffee with a mellow and long aftertaste (24). Protein contents generally impact Maillard reaction and caramelization, thus affecting the formation of coffee flavor substances and the sensory quality of coffee, such as color, taste, and aroma. Franca et al. (25) showed that high-quality coffee beans have a higher protein content, while Macrae (26) suggested no necessary relationship between protein content and coffee flavor quality. To better display multidimensional data, a spider plot was also used to visually show the main physicochemical characteristics and the EY obtained by different brewing methods (Figure 1C). Among the three cold-brewing methods, the EY of UAC was slightly higher than that of the other two counterparts, which demonstrated that ultrasound directly acts on mass transfer improvement and impacts positively on the basic mechanisms of extractions: desorption and diffusion of a solute out of the raw material structure (27). The ultrasonic capillary effect could also explain the enhanced extraction effect, but its detailed mechanism is not fully understood (10). Overall, regarding extraction ability and general physicochemical characteristics in coffee, UAC was an effective cold brewing method, even better than static cold extraction at 4 and 10°C.

### Effects of Hot- and Cold-Brewing on Caffeine, CGA, and Trigonelline Contents

The most abundant alkaloids in coffee are caffeine and trigonelline, vital to the coffee flavor. CGA is also present in coffee, with relatively high amounts contributing to the health benefits. Therefore, HPLC was adopted in their analysis to clarify the differences between hot and cold brews. The hot brewing methods produced higher caffeine, CGA, and trigonelline contents (Table 2), according to linear regression equations (Supplementary Table 2). Caffeine has a limited solubility at an ambient temperature of 16 mg/mL (8) and is less sensitive to roasting temperature than CGA. This may explain why 4 and 10 CB exhibited similar caffeine concentrations. CGA, unlike caffeine, is freely soluble in water (8), and this facilitates its extraction by both hot and cold brewing methods. Concentrations were the highest in PO coffees (1.53 mg/mL for CGA and 0.54 mg/mL for trigonelline) because the dynamic PO method (add water in three stages) involved the continuous renewal of the extraction. Compared to a static system, this is definitely a more efficient way of extracting relevant molecules (6). Fuller et al. (8) have investigated the extraction kinetics and equilibrium concentrations of caffeine and CGA in cold brew coffee and proved that caffeine and CGA reached nearly steady-state concentrations after 400 min. Since extraction time in UAC was only 60 min, the contents of caffeine and CGA obtained were significantly lower than that of static cold brews (12 h). Although CGA and its derivatives are important flavor-regulating substances of coffee, they also contribute to the acidity, astringency, and bitterness of the final coffee beverages (6). Also, caffeine and trigonelline represent a maximum of 10–30% and 1% of the total bitter taste intensity, respectively (28). Many researchers considered that CGA, trigonelline, and caffeine's degradation products were related to the bitterness of coffee beverages (29). Therefore, UAC coffee with relatively lower CGA (1.25 mg/mL) and trigonelline (0.42 mg/mL) contents may make it less bitter than other coffees. Similarly, the previous study has described that over-brewing cold brew coffee may result in unpalatable flavor due to degradation of CGA and other relatively slow-extracting compounds such as 4-vinylcatechol oligomers (30). Angeloni et al. (6) also ascribed the higher concentrations of caffeine and CGA to the prolonged contact time during conventional cold brewing compared to hot brewed coffees.

**Table 2:** Contents of caffeine, CGA, and trigonelline in coffee with different brewing methods.

**Brewing methods**		**Extraction conditions**	**Caffeine** **(mg/mL)**	**CGA** **(mg/mL)**	**Trigonelline (mg/mL)**
**Hot brewing**	HB	95°C, 5 min	0.64 ± 0.00^b^	1.29 ± 0.00^c^	0.49 ± 0.00^c^
	PO	92°C, 3 min (three-stage)	0.68 ± 0.00^a^	1.53 ± 0.05^a^	0.54 ± 0.00^a^
**Cold brewing**	4 CB	4°C, 12 h	0.60 ± 0.00^c^	1.37 ± 0.00^b^	0.50 ± 0.00^b^
	10 CB	10°C, 12 h	0.60 ± 0.00^c^	1.35 ± 0.04^b^	0.31 ± 0.00^e^
	UAC	200 W, RT, 60 min	0.56 ± 0.00^d^	1.25 ± 0.00^c^	0.42 ± 0.00^d^

*HB, Hot boiled; PO, Pour-over; 4CB, 4°C cold brew; 10CB, 10°C cold brew; UAC, Ultrasound-assisted cold brew; CGA, Chlorogenic acid. Different letters indicate statistically significant differences (P < 0.05) among treatments*.

### Volatile Compound Profiles of Hot and Cold Brew Coffees

Flavor is a key attribute in determining coffee products' quality and consumer acceptance. The volatiles present in the coffee brew depends on the extraction technique. Therefore, in the present study, HS-SPME-GC/MS was used for qualitative and quantitative analysis of volatile components in hot and cold brew coffees (Table 3). Total ion chromatograms of volatiles with different methods are shown in Supplementary Figure 1.

**Table 3:** Volatile components in coffee extracts with different brewing methods.

**NO**.	**Category**	**CAS**	**Compounds**	**Contents (μg/mL)**				
				**HB**	**PO**	**4 CB**	**10 CB**	**UAC**
1	Furans	3188-00-9	2-Methyl tetrahydro-3-furanone	0.44	0.51	0.30	0.21	0.13
2		4412-91-3	3-Furyl alcohol	14.95	16.21	10.86	9.13	7.26
3		1192-62-7	2-Acetylfuran	2.56	2.25	1.47	1.16	0.83
4		4466-24-4	2-N-butyl furan	1.06	0.66	0.50	—	0.25
5		620-02-0	5-Methylfuran aldehyde	21.31	20.89	10.52	9.09	6.35
6		3194-15-8	2-Propionylfuran	1.01	0.52	—	0.23	0.22
7		5989-33-3	Cis-α,α-5-trimethyl-5-vinyl tetrahydrofuran-2-methanol	2.96	2.03	1.54	0.37	0.78
8		10599-69-6	2-Methyl-5-propionylfuran	—	—	—	0.16	0.12
9		31681-26-2	α -Propyl-2-furan acetaldehyde	0.73	0.71	—	—	—
10		1193-79-9	5-Methyl-2-acetyl-furan	1.12	1.69	—	0.89	0.61
11		19377-82-3	2-N-(furanyl) furosemide	—	1.44	—	—	—
12		98-01-1	Furfural	8.35	10.68	5.02	3.86	2.60
13		488-05-1	3-Methyl-1- (3-methyl-2-furanyl)−1-butanone	—	0.55	—	—	—
14		3777-71-7	2-Heptyl furan	—	—	0.37	0.21	0.15
15		3208-16-0	2-Ethylfuran	—	—	0.80	0.49	0.30
16		10410-20-5	2-(1-Hydroxy-1-methyl-2-oxo-propyl)-2, 5-dimethylfuran-3 (2H) -ketone	—	—	—	0.17	0.13
17	Pyrazines	71257-37-9	3, 4-Dihydropyrrole and [1,2-a] pyrazine	—	—	—	0.28	—
18		5910-89-4	2,3-Dimethylpyrazine	0.38	0.35	0.21	0.11	0.13
19		23747-48-0	5-Methyl-6,7-dihydro-5H-cyclopentaminopyrazine	0.49	0.52	0.37	0.27	0.19
20		22047-26-3	2-Acetyl-6-methylpyrazine	0.32	0.30	—	0.20	0.16
21		18217-82-8	2-Methyl-5-[(E)-1-allyl] pyrazine	0.32	0.46	0.34	0.21	—
22		14667-55-1	2,3,5-Trimethylpyrazine	—	7.50	3.97	2.65	2.05
23		13925-09-2	2-Vinyl-6-methylpyrazine	—	—	—	0.28	—
24		13925-08-1	2-Vinyl-5-methylpyrazine	—	0.22	—	—	—
25		13925-07-0	2-Ethylalkyl-3,5-dimethylpyrazine	—	1.67	—	—	—
26		13925-03-6	2-Ethyl-6-methylpyrazine	5.52	4.43	2.84	1.99	1.48
27		13925-00-3	2-Ethylpyrazine	2.05	1.13	0.74	0.64	0.48
28		13360-65-1	3-Ethyl-2,5-methylpyrazine	5.54	4.20	3.22	1.80	1.39
29		109-08-0	2-Methylpyrazine	3.04	3.96	1.99	1.47	1.05
30		108-50-9	2, 6-Dimethylpyrazine	9.29	9.53	4.84	3.71	2.61
31		15707-23-0	2-Ethyl-3-methylpyrazine	13.64	—	—	—	—
32		98-96-4	Pyrazinamide	—	—	—	0.14	0.10
33	Alcohols	98-55-5	Alpha terpineol	—	0.25	0.30	0.14	—
34		78-70-6	Linalool	0.84	0.30	0.57	0.20	0.19
35		768-95-6	1-Adamantanol	0.94	0.92	0.63	0.40	0.29
36		60-12-8	Phenethyl alcohol	—	—	—	0.64	0.48
37	Alcohols	59-42-7	3-Hydroxyl-α-(methyl amino) methyl benzyl alcohol	—	0.20	—	—	—
38		64-17-5	Ethyl alcohol	0.33	—	—	—	—
39		111-55-7	Glycol diacetate	0.71	—	—	—	—
40		543-49-7	2-Heptanol	—	—	0.24	—	—
41		14003-34-0	3-Methyl-3-quinolol	—	—	—	0.14	—
42		1075-04-3	1-Phenylpropane-1,2-diol	—	—	—	0.93	0.68
43	Pyrroles	86688-96-2	(Pyrrole-3-yl)-acetic acid	—	—	1.22	0.41	0.27
44		2167-14-8	Tea pyrrole	0.16	—	—	—	—
45		19713-89-4	3, 4-Dimethyl-1-H-pyrrol-2-carboxyaldehyde	1.43	—	0.40	0.26	0.31
46		1192-58-1	N-methyl-2-pyrrole formaldehyde	—	4.32	2.99	2.35	1.60
47		1072-83-9	2-Acetyl-pyrrole	2.74	0.85	1.10	1.85	1.32
48		1438-94-4	1-Furfuryl pyrrole	—	—	0.47	—	0.26
49		1003-29-8	2-Pyrrolidoformaldehyde	—	—	0.24	—	0.28
50	Aldehydes	96-17-3	2-Methylbutylaldehyde	0.56	0.52	0.50	0.16	0.14
51		78-84-2	Isobutyraldehyde	—	0.28	—	—	—
52		590-86-3	Isovaleraldehyde	0.40	0.32	0.38	0.09	0.08
53		122-78-1	Phenylacetaldehyde	0.44	0.22	—	—	0.10
54	Ketones	875-59-2	4-Hydroxy-2-methyl acetophenone	6.84	6.82	3.49	2.31	1.46
55		75-97-8	Tert-butyl methyl ketone	—	—	—	0.54	—
56		600-14-6	2,3-Glutaric ketone	0.78	1.10	0.49	0.22	0.16
57	Ketones	592-20-1	Acetoxy-2-acetone	—	1.00	0.52	0.30	0.39
58		1575-57-1	1-Acetoxy-2-butanone	—	0.38	0.19	0.15	0.16
59		67-64-1	Acetone	—	0.44	—	—	—
60		21835-01-8	3-Ethyl-2-hydroxy-2-cyclopentene-1-ketone	—	0.68	—	—	—
61		765-70-8	3-Methylcyclopentane-1,2-dione	0.69	—	—	—	—
62		699-83-2	2,6-Dihydroxyacetophenone	—	—	2.71	—	1.22
63		34598-80-6	2, 4-Dimethylcyclopentane-1,3-dione	—	—	1.21	—	—
64		5751-48-4	2-Methylchromone	—	—	—	0.14	—
65	Phenols	95-48-7	2-Cresol	—	—	0.51	—	0.39
66		90-05-1	Guaiacol	2.54	4.92	1.94	1.89	1.77
67		2785-89-9	4-Ethyl-2-methoxyphenol	3.71	3.00	—	2.66	—
68		108-95-2	Phenol	—	—	—	1.19	0.89
69		2836-00-2	3-Amino-4-methylphenol	—	1.20	—	—	—
70		1138-52-9	3, 5-Di-tert-butylphenol	—	0.67	—	0.37	—
71		106-44-5	Paracresol	—	1.13	—	0.37	—
72	Pyridines	110-86-1	Pyridine	16.63	12.10	11.16	4.06	4.11
73		67402-83-9	1-Acetyl-1,4-dihydropyridine	0.33	0.30	—	—	—
74		17747-43-2	3-Acetoxyridine	—	0.16	—	—	—
75	Acids	503-74-2	Isopropylaceticacid	0.90	—	1.40	—	—
76		53387-38-5	Cyclohexylacetic Acid	1.29	—	—	0.29	—
77		631-61-8	Ammonium acetate	0.27	—	—	—	—
78	Esters	623-17-6	Furfuryl acetate	—	—	—	—	0.31
79		547-65-9	2-Methylenyl butyrolactone	—	0.22	—	0.17	—
80		62873-16-9	α -methylene-γ-valerolactone	—	0.32	—	—	—
81		36760-43-7	S-phenyl-tert-butyl thiocarbonate	1.47	1.17	0.57	—	0.43
82		68555-59-9	α,2, 2, 6-Tetramethylcyclohexyl acetate, propyl	0.23	—	—	—	—
83		1129-41-5	1-Acetoxy-2-butanone	1.34	—	—	—	—
84		79-20-9	Acetone	—	—	0.35	—	—
85		6203-89-0	3-Ethyl-2-hydroxy-2-cyclopentene-1-ketone	—	—	0.42	—	—
86		13290-00-1	3-Methylcyclopentane-1, 2-dione	—	—	0.36	—	—
87		7779-73-9	2, 6-Dihydroxyacetophenone	—	—	—	0.25	—
88		103130-72-9	2, 4-Dimethylcyclopentane-1, 3-dione	—	—	—	—	0.08
89	Others	96-37-7	2-Methylchromone	1.82	1.12	0.89	0.33	0.99
90		6596-35-6	Four hydrogen naphthalene anthracene	0.72	0.38	0.32	—	0.28
91		6380-23-0	3, 4-Dimethoxy styrene	0.84	0.55	0.48	0.20	—
92		491-36-1	4-Hydroxyquinazoline	—	0.56	0.50	0.23	0.19
93		4634-87-1	2, 6-Dimethyl-2, 4-heptadiene	—	—	—	0.26	0.24
94		4437-22-3	2, 2-Difuryl ether	1.19	0.73	0.82	0.53	0.24
95		3891-99-4	2,6, 10-Trimethyltridecane	0.40	0.22	0.28	0.07	0.12
96		1921-70-6	2,6,10, 14-Tetramethylpentadecane	—	0.62	0.36	—	0.16
97		55401-75-7	9-Dodecyl tetoxanthracene	0.97	—	0.51	—	—

*HB, Hot boiled; PO, Pour-over; 4CB, 4°C cold brew; 10 CB, 10°C cold brew; UAC, Ultrasound-assisted cold brew*.

### Qualitative Analysis

**Figure 2:**
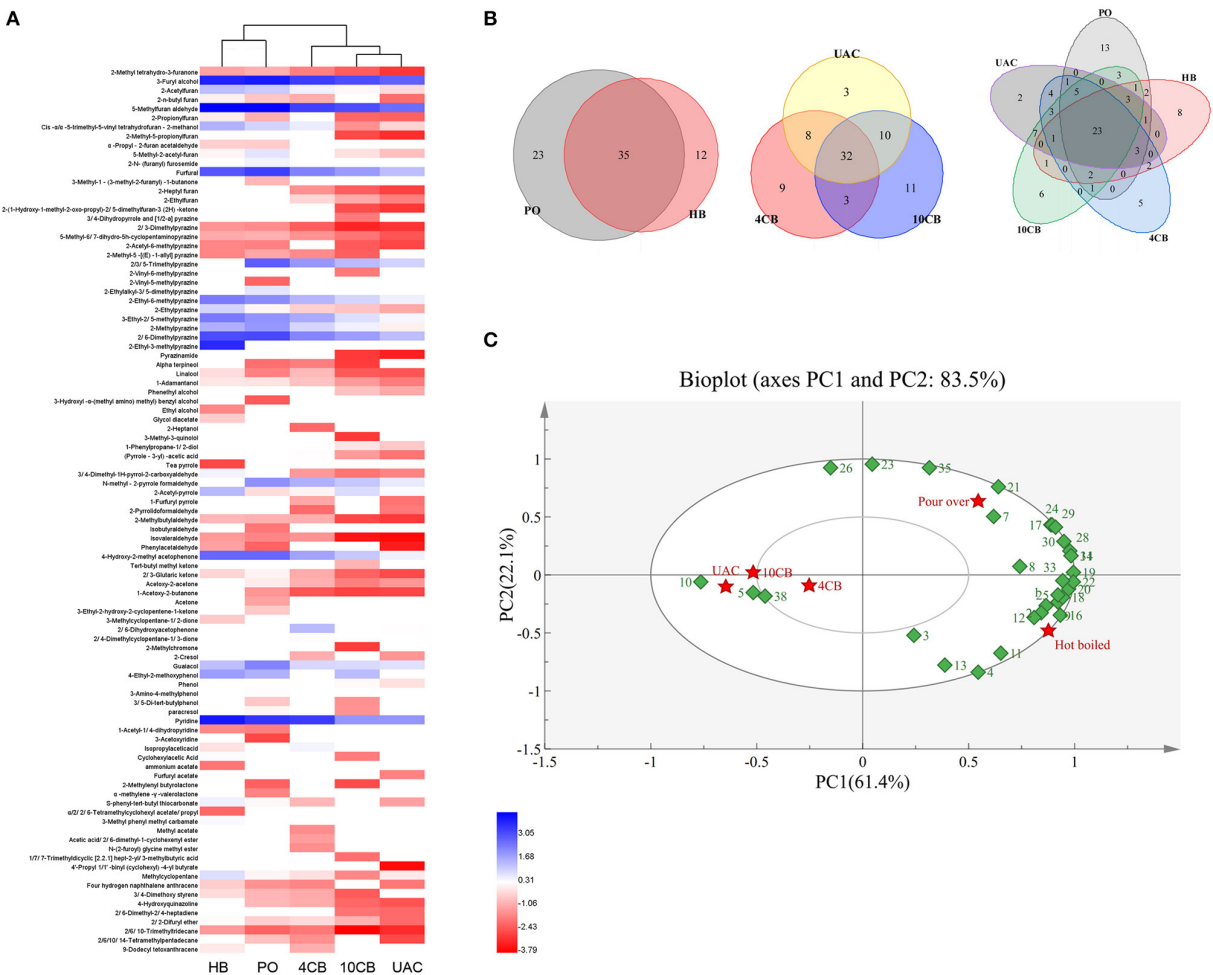
Cluster heatmap **(A)** and Venn diagrams **(B)** of volatile compounds produced by different coffee brewing methods. **(C)** Principal component analysis (PCA) biplot of volatile compounds in hot and cold brew coffees. (1: 2-Propionylfuran, 2: 2, 2-Difuryl ether, 3: Isopropylacetic acid, 4: 3, 4-Dimethyl-1H-pyrrol-2-carboxyaldehyde, 5: (Pyrrole-3-yl) -acetic acid, 6: 2-N-(furanyl) furosemide, 7: 5-Methyl-2-acetyl-furan, 8: 4-Ethyl-2-methoxyphenol, 9: 2-n-butyl furan, 10: Phenol, 11: Cyclohexylacetic Acid, 12: Methylcyclopentane, 13: 2-Acetyl-pyrrole,14: 4-Hydroxy-2-methyl acetophenone, 15: 2-Ethylalkyl-3, 5-dimethylpyrazine, 16: 2-Ethylpyrazine, 17: 2, 3-Pentanedione, 18: Cis -α,α−5-trimethyl-5-vinyl tetrahydrofuran-2-methanol, 19: 2-Acetylfuran, 20: 3-Ethyl-2, 5-methylpyrazine, 21: Guaiacol, 22: 2-Ethyl-6-methylpyrazine, 23: 2,3,5-Trimethylpyrazine, 24: 2-Methylpyrazine, 25: Pyridine, 26: N-methyl-2-pyrrole formaldehyde, 27: Furfuryl acetate, 28: 2, 6-Dimethylpyrazine, 29: Furfural, 30: 3-Furyl alcohol, 31: 5-Methylfuran aldehyde, 32: 3-Methyl phenyl methyl carbamate, 33: S-phenyl-tert-butyl thiocarbonate, 34: 2-Ethyl-3-methylpyrazine, 35: Paracresol, 36: 3-Amino-4-methylphenol, 37: 2, 4-Dimethylcyclopentane-1, 3-dione, 38: 2,6-Dihydroxyacetophenone). HB, Hot boiled, PO, Pour-over, 4 CB, 4°C cold brew, 10 CB,10°C cold brew, UAC, Ultrasound-assisted cold brew.

A total of 97 volatile compounds were identified in hot and cold coffee brews, including furans (16), pyrazines (16), alcohols (10), pyrroles (7), aldehydes (4), ketones (11), phenols (7), pyridines (3), acids (3), esters (11), and others (9). Lopez-Galilea et al. (31) have reported that pyrazines, furans, aldehydes, and ketones have a high impact on the aroma of coffee. The current results showed that most of the volatile compounds identified in hot and cold coffee extractions belonged to these chemical classes. For the cold brews, 53 volatile compounds were found in UAC coffee, between 4 CB (52) and 10 CB (56). Three special volatiles were found in the UAC coffee, including phenylacetaldehyde, 4′-propyl-1,1′-biphenyl (cyclohexyl)-4-butyrate, and furfuryl acetate, which belonged to aldehydes and esters. Aldehydes have been described as having chocolate and malty odors (1), and esters are related to fruity flavor in coffee (32). Both roasting process and extraction technology influence the presence of volatile compounds in the final coffee. Venn diagrams (Figure 2B) clearly showed the volatiles in different groups. Although cold-brew coffee's overall volatiles were less than their hot counterparts, the cold coffee beverages had a unique and acceptable flavor.

The abundance of 97 volatile compounds in hot and cold coffee extractions was analyzed by hierarchical clustering (Figure 2A). The coffee samples were divided into two groups, where HB and PO coffee were classified as one group. Both were performed at relatively high temperatures, contributing to more volatiles, especially furans and pyrazines. Cold-brew extractions were divided into a second group, and the volatiles between 10 CB and UAC were more similar. To further clarify the effects of different extraction methods on volatile components, the main volatiles (content greater than 0.1 μg/mL) were selected for principal component analysis (PCA). As shown in Figure 2C, two principal components represented more than 83.5% of the total variance in the volatiles, and PC1 and PC2 of the PCA analysis model explained 61.4 and 22.1% of the variance, respectively. The hot and cold brewing methods were clearly separated by PC1, indicating the main volatile compounds differed between hot and cold counterparts. Phenols, ketones, and furans, such as guaiacol, 2,3-pentanedione, and 5-methyl-2-acetylfuran, were grouped in the first quadrant with PO coffees. While pyrazines and acids, including 2-ethyl-3-methylpyrazine, 2-ethylpyrazine, and cyclohexanacetic acid, were grouped in the fourth quadrant with HB extractions, most of the volatiles correlated with 4 CB, 10 CB, and UAC coffee beverages were concentrated in the negative axis of PC1, such as furfuryl acetate and some ketones derivatives, further indicating that there was no significant difference in the aroma and flavor among cold brew coffees, which was consistent with cluster analysis results (Figure 2A).

### Quantitative Analysis

**Figure 3:**
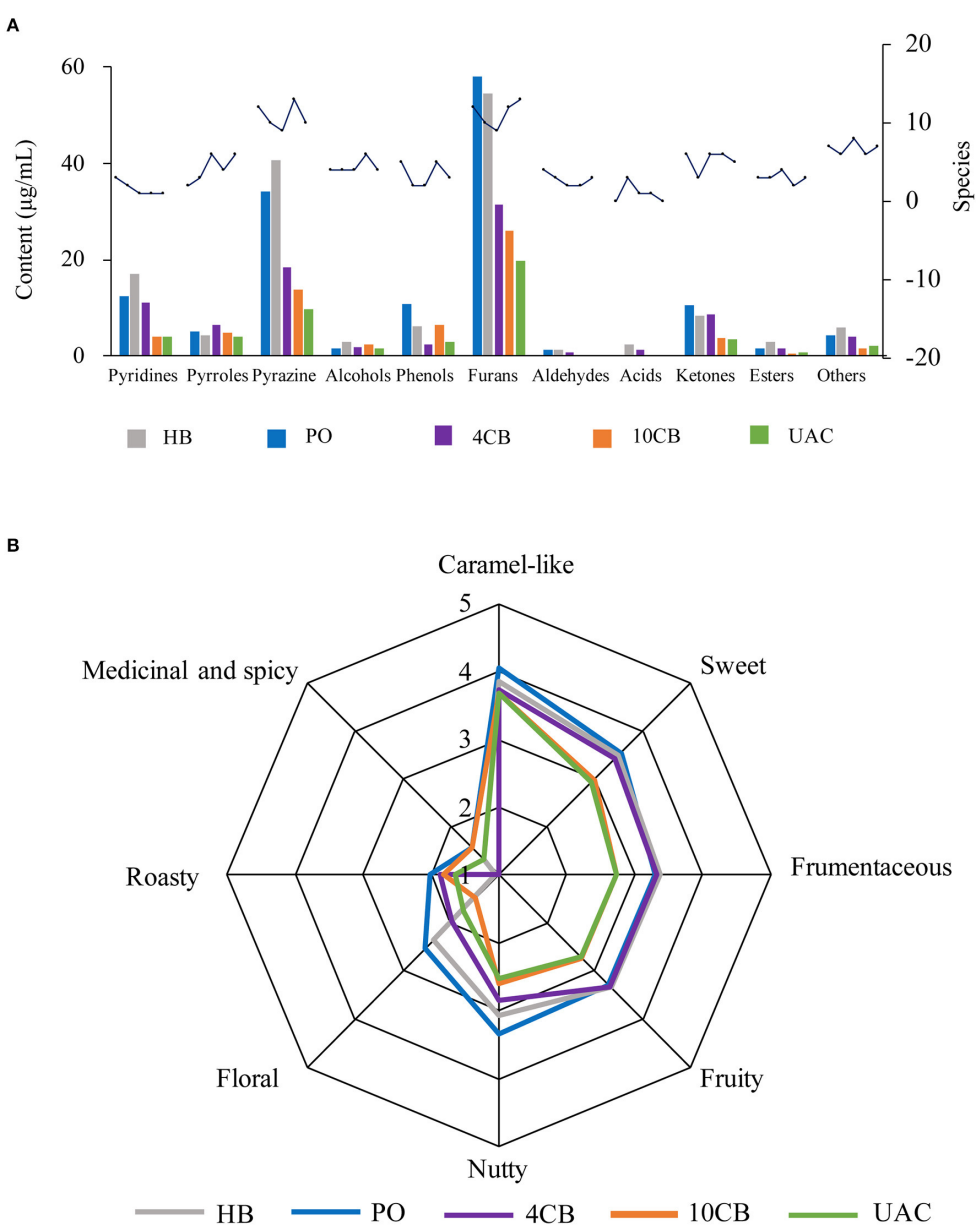
Contents and species of volatile compounds **(A)** and spider plots of aroma attributes **(B)** for different coffee brewing methods. HB, Hot boiled, PO, Pour-over, 4 CB, 4°C cold brew, 10 CB, 10°C cold brew, UAC, Ultrasound-assisted cold brew.

The species and contents of the volatile compounds were identified in hot and cold brew coffees by HS-SPME-GC/MS, and are presented in Figure 3A and Table 3. It was observed that the volatile profiles of different extractions were generally similar. Furans were the most abundant class of volatiles detected in coffees, which could be formed through thermal degradation of carbohydrates, ascorbic acid, or unsaturated fatty acids during coffee roasting (33). UAC extractions contained 19.73 μg/mL furans, accounting for 40.31% of the total volatiles, and were slightly lower than that of 4 CB (31.38 μg/mL) and 10 CB (25.94 μg/mL). Compared to other volatiles in coffee, furans had relatively high thresholds and mainly exhibited malty and sweet roasted aromas. 3-Furan methanol, 5-methylfuran aldehyde, and furan formaldehyde were found in large amounts in UAC coffees, associated with almond notes, caramel, and burnt sugar.

Pyrazines had the second-highest number of volatile compounds in all samples, including UAC coffee. They are mainly generated by Strecker degradation of aldehydes interacting with amino ketones provided by amino acids (34). The contents of pyrazine compounds in PO and HB coffee were approximately 2 times the cold brews, indicating more potent nutty and roasty aromas in hot brew coffees. The contents of pyrazines in UAC extractions were 9.64 μg/mL (19.69%), and 2,6-dimethylpyrazine and 2-ethyl-6-methylpyrazine were more abundant.

Heterocyclic compounds, such as pyridines and pyrroles, were also detected in hot and cold brew coffees. In general, pyridines in coffee mainly include alkylpyridine and acylpyridene, which endow coffee roasting/burnt flavor and biscuit aroma, respectively. Acylpyridine was only identified in hot brew coffee samples, indicating a unique biscuit aroma. Pyrroles have been reported as furan degradation products and amino acid derivatives (2), responsible for a peculiarly sweet and slightly flared smell in coffee (35). Pyridines and pyrroles showed low concentrations in UAC coffees (4.11 μg/mL and 4.04 μg/mL, respectively), indicating a relatively weak burnt and smoky aroma.

There were also some ketones and phenols present in coffees. Ketones are known to be responsible for buttery, caramel-like, or fruity odor notes (31), and phenols have been described as having smoky, spicy, and burnt aromas (36). The content of ketones in UAC extractions was the lowest (3.39 μg/mL), which was about one-third of that in PO coffee. In contrast, phenols were 3.05 μg/mL in content, slightly higher than 4 CB. The UAC method demonstrated the low extraction ability of ketones and phenols in coffee.

Table 4 shows the compounds with OAV values greater than 1, and the sensory descriptors and the odor perception threshold values were taken from the literature results (31, 37). Twenty-nine compounds were determined in five kinds of coffees at concentrations higher than their corresponding odor threshold value, including pyrazines (6), furans (5), phenols (5), aldehydes (4), ketones (2), alcohols (2), pyrroles (2), pyridines (2), and ester (1), which possibly contributed to the overall coffee aroma. According to Table 4, the aromas can be summarized into eight categories: caramel-like, sweet, frumentaceous, fruity, nutty, floral, roasty, medicinal, and spicy aroma (Figure 3B), which revealed apparent differences in the aroma profiles of the respective extraction methods. Caramel-like is the strongest aroma, which was mainly contributed by guaiacol (OAV > 3000) and 3-ethyl-2,5-dimethylpyrazine (OAV > 200). According to Semmelroch & Grosch (36), these two compounds played an essential role in the aroma of coffees. Sweet, frumentaceous, fruity, and nutty aromas were also abundant in coffees, mainly owing to isovaleraldehyde (OAV > 400) and some pyrazine derivatives. While floral, roasty, medicinal, and spicy aroma had relatively little effect on flavor, On the whole, the aroma of coffee extracted by UAC might be less intense than that of hot extractions, but was similar to that of 10 CB extractions.

**Table 4:** Odor thresholds and odor activity values (OAVs) of potent volatile compounds in coffee extracts with different brewing methods.

**NO**.	**Compounds**	**Odor descriptors**	**Odor threshold (μg/L)**	**Odor activity value (OAV)**				
				**HB**	**PO**	**4 CB**	**10 CB**	**UAC**
1	3-Furyl alcohol	Peculiar bitter, spicy	2,000	7.47	8.10	5.43	4.56	3.63
2	2-N-butyl furan	Wine, bread, meat	5	211.60	132.83	100.10	—	49.53
3	5-Methylfuran aldehyde	Caramel	1,110	19.20	18.82	9.48	8.19	5.72
4	2-Ethylfuran	Sweet	2.3	—	—	345.65	211.07	131.72
5	2-Acetyl-6-methylpyrazine	Roasty, nutty	62	5.18	4.91	—	3.17	2.56
6	2,3, 5-Trimethylpyrazine	Nutty, sweet, floral	1,800	—	4.17	2.21	1.47	1.14
7	2-Ethylalkyl-3,5-dimethylpyrazine	Nutty, sweet	1	—	1,671.63	—	—	—
8	2-Ethyl-6-methylpyrazine	Caramel, nutty	100	55.15	44.27	28.44	19.92	14.85
9	3-Ethyl-2,5-methylpyrazine	Almond, chocolate, caramel	5	1,107.73	839.21	644.38	360.86	277.22
10	2, 6-Dimethylpyrazine	Roasty, chocolate	9,000	1.03	1.06	0.54	0.41	0.29
11	2-Ethyl-3-methylpyrazine	Nutty, chocolate, peanut	130	104.92	—	—	—	—
12	Linalool	Floral, spices, wood	6	140.41	50.80	95.81	32.80	31.12
13	2-Heptanol	Lemon, grassy	65.2	—	—	3.61	—	—
14	N-methyl - 2-pyrrole formaldehyde	Roasty, almond	37	—	116.86	80.73	63.59	43.25
15	1-Furfuryl pyrrole	Hazelnut, cocoa	100	—	—	4.70	—	2.62
16	2-Methylbutylaldehyde	Apple, malt, and fermented	1.3	432.44	403.40	386.37	123.11	105.77
17	Isobutyraldehyde	Fruity, malt, and grassy	0.9	—	306.57	—	—	—
18	Isovaleraldehyde	Apple, chocolate, cheesy, sweet	0.2	1,998.30	1,619.64	1,894.72	465.63	402.77
19	Phenylacetaldehyde	Sweet, grassy, floral, chocolate	4	109.40	55.30	—	—	24.93
20	2, 3-Glutaric ketone	Creamy	30	26.03	36.59	16.33	7.22	5.48
21	3-Furyl alcohol	Caramel, nutty, bread	300	2.31	—	—	—	—
22	2-N-butyl furan	Caramel	0.381	6,666.67	12,919.95	5,104.40	4,973.02	4,652.80
23	5-Methylfuran aldehyde	Spicy, smoky	540	6.88	5.55	—	4.93	—
24	2-Ethylfuran	Phenolic, medicinal	58.6	—	—	—	20.24	15.19
25	2-Acetyl-6-methylpyrazine	Alkylphenolic	200	—	3.35	—	1.83	—
26	2,3, 5-Trimethylpyrazine	Medicinal, phenolic	55	—	20.46	—	6.81	—
27	2-Ethylalkyl-3, 5-dimethylpyrazine	Special smell, pungent	2,000	8.32	6.05	5.58	2.03	2.05
28	2-Ethyl-6-methylpyrazine	Nutty	32.19	—	4.97	—	—	—
29	3-Ethyl-2, 5-methylpyrazine	Fruity	1,030	—	—	—	—	0.30

*HB, Hot boiled; PO, Pour-over; 4 CB, 4°C cold brew; 10 CB,10°C cold brew; UAC, Ultrasound-assisted cold brew*.

### Optimization of UAC Coffee

Compared to hot-brewing, the coffee produced by cold-brewing has a unique sensory flavor and good market prospect. However, the problems of long extraction time and high energy consumption limit the large-scale production of cold-brewing. The above results showed that UAC considerably shortened cold extraction time (1 h vs. 12 h) and made main physicochemical characteristics achieve the levels of coffee produced through conventional cold-brewing methods. The aroma was similar to that of 10 CB coffee. Therefore, UAC can replace conventional cold extractions, significantly improving the extraction efficiency and ensuring the quality of coffee.

The coffee-to-water ratio is significantly associated with mass transfer during the extraction process. Studies show that using a lower coffee/water ratio decreases the titratable acidity/total polyphenol concentration ratio and changes the coffee flavor (38). While a high amount of coffee decreases bed permeability, resulting in excessive pressure and possible over extraction (17), EY can be defined as a specified component released from the coffee matrix at a given time (19). So extraction time is another crucial factor for coffee flavor and quality. Likewise, ultrasound has been proposed as an effective technology to reach higher EY than conventional extraction methods due to faster extraction kinetics, with ultrasonic power as the most critical parameter. Therefore, the UAC conditions, including coffee/water ratio, extraction time, together with ultrasonic power, were optimized by OED.

Based on the optimal conditions obtained from single-factor experiments [coffee-to-water ratio, 1:15; extraction time, 60 min; ultrasonic power, 150 W (Supplementary Figure 2)], the OED L_9_ (3^3^) was employed to investigate the effects of coffee/water, extraction time, and ultrasonic power. The details of the OED are shown in Supplementary Table 3. The K values (K1, K2, and K3) were the average sum of indicators at each factor level, and the R-value was the influence of the experimental parameters. Higher R indicates the factor has a more decisive influence. Therefore, the optimal conditions were coffee/water = 1:15, extraction time, 60 min, with ultrasonic power 200 W, leading to the increased EY, high to 17.72%. Using the OED substantially reduced the number of experiments required, saving the time and cost spent on conducting experiments.

After that, the physicochemical characteristics, including TDS, TPC, TO, Tpro, TA, pH, color, and main no-volatiles including caffeine, CGA, and trigonelline contents, were evaluated under optimal condition (Supplementary Table 4). Compared to unoptimized UAC coffee, almost all component extraction rates increased, while L^*^ value and CGA content slightly decreased, indicating darker color with a lesser bitter taste in UAC coffee after optimization.

## Conclusions and Perspectives

Cold brew coffee is rapidly growing, despite the lack of a standardized procedure for its production. In this work, cold brewing (immersion at 4°C/10°C and UAC) and hot brewing (HB and PO) extractions with the same coffee-to-water ratio were performed to identify any differences concerning the contents of physicochemical characteristics, mainly non-volatile components and volatiles. Results showed that the traditional cold-brewing method could be optimized by sonication, leading to shorter extraction time and improved extraction efficiency. Based on our data, the UAC-generated beverages displayed higher TDS, TL, Tpro, TA, and pH than static cold immersion coffee, with comparable contents of caffeine, CGA, and trigonelline. The superior quality aroma of coffee appeared to be related to well-defined temperature and pressure parameters because compounds with high polarity, such as alcohols, ketones, pyrazines and aldehydes, tend to percolate more quickly in the greatest abundance contributing to the potency and intensity of the coffee aroma in hot extractions. UAC coffee was mainly associated with flavor attributes such as caramel, nutty, roasty and sweet aromas, which had no significant difference from traditional cold brews. Further research is needed to characterize the UAC coffee by its sensory evaluation and consumer acceptability. Another vital issue that must be considered is the safety of cold brew coffee. Due to the long-brewing time for conventional cold brewing methods, it tends to facilitate the activity of microorganisms to cause microbiological food safety hazards. However, few published studies focused on the safety of cold coffee brews. Recently, Kyroglou (4) mentioned that various species of pathogenic bacteria might be viable in the cold brew for 7–28 days. Sonication is considered a non-thermal food preservation method that can inactivate microorganisms, so UAC may have an additional and surprising effect on cold brew coffee's bacteriostasis, which needs further research.

On the whole, with the optimum conditions for UAC, a new cold brew method was established, which can reduce the extraction time and improve extraction efficiency, with a similar aroma and flavor to original cold brew coffee. This study attempts to contribute to the processing of cold brew coffee, bringing in a promising fast extraction procedure that will shed light on the commercialization of the process. Future work should be conducted to evaluate other parameters for UAC; such as species of coffee beans, degree of grinding, roasting, and temperature; and the safety issues concerning cold coffee brew consumption, which will promote completing the work for industrial application of UAC brewing coffee.

## Data Availability Statement

The original contributions presented in the study are included in the article/Supplementary Material, further inquiries can be directed to the corresponding author/s.

## Author Contributions

XZ conducted most of the statistical analyses, wrote, and edited the manuscript. MY and JZ conducted the research. LZ provided expertise regarding the volatiles aspects of the work. YT and CL completed the optimization of UAC coffee. LB helped analyze the volatiles data. CM designed the experiments and provided consultation in terms of chemical and physical analyses. AA formal analysis, writing–review & editing. All authors contributed to this work. All authors contributed to the article and approved the submitted version.

## Funding

The work was supported by Fundamental Research Funds for the Central Universities, China (2021ZY66), and Postdoctoral Research Foundation of China (2021M690420).

## Conflict of Interest

The authors declare that the research was conducted in the absence of any commercial or financial relationships that could be construed as a potential conflict of interest.

## Publisher's Note

All claims expressed in this article are solely those of the authors and do not necessarily represent those of their affiliated organizations, or those of the publisher, the editors and the reviewers. Any product that may be evaluated in this article, or claim that may be made by its manufacturer, is not guaranteed or endorsed by the publisher.

## Abbreviations

HB: Hot boiled method
PO: Pour-over method
4CB: 4°C cold brewing method
10CB: 10°C cold brewing method
UAC: Ultrasound-assisted cold brewing method
EY: Extraction yield
TDS: Total dissolved solids
TL: Total lipid extraction rate
TPC: Total phenolic extraction rate
Tpro: Total protein extraction rate
TA: Total acid extraction rate
CGA: Chlorogenic acid.
