# Supplementary material for: Feasibility of Ultrasound-Assisted Extraction for Accelerated Cold Brew Coffee Processing: Characterization and Comparison With Conventional Brewing Methods
Source: Front Nutr. 2022 Mar 18;9:849811. doi: 10.3389/fnut.2022.849811 (PMC8973412; doi:10.3389/fnut.2022.849811)
Supplement: Supplementary file 1 [file Data_Sheet_1.docx]

**Table S1**. Comparison of coffee physicochemical characteristics using hot and cold brewing methods.

| **Brewing method** | | **Extraction conditions** | **EY (%)** | **TDS (°Brix)** | **pH** | **Color** | | | **TPC (%)** | **TL (%)** | **Tpro (%)** | **TA (%)** |
| --- | --- | --- | --- | --- | --- | --- | --- | --- | --- | --- | --- | --- |
|  |  |  |  |  |  | ***L**** | ***a**** | ***b**** |  |  |  |  |
| **Hot brewing** | HB | 95℃, 5 min | 18.78±0.29^a^ | 1.15±0.10^a^ | 5.18±0.02^d^ | 2.21±0.31^a^ | -0.01±0.04^b^ | 0.38±0.06^b^ | 2.54±0.06^a^ | 4.69±0.31^a^ | 3.38±0.09^a^ | 0.88±0.02^a^ |
|  | PO | 92℃, 3 min (three-stage) | 18.69±0.63^a^ | 0.91±0.12^c^ | 5.09±0.01^c^ | 2.14±0.49^a^ | -0.09±0.02^c^ | 0.34±0.02^b^ | 2.16±0.04^b^ | 3.54±0.33^c^ | 2.85±0.08^c^ | 0.80±0.03^b^ |
| **Cold brewing** | 4CB | 4℃, 12 h | 16.22±0.65^c^ | 0.98±0.12^bc^ | 5.19±0.0^bc^ | 2.96±0.57^a^ | -0.01±0.01^b^ | 0.38±0.06^b^ | 1.83±0.12^c^ | 4.25±0.12^ab^ | 2.51±0.04^d^ | 0.72±0.04^c^ |
|  | 10CB | 10℃, 12 h | 15.81±0.30^c^ | 1.17±0.12^a^ | 5.21±0.0^b^ | 3.14±0.42^a^ | -0.12±0.01^c^ | 0.48±0.05^a^ | 1.83±0.04^c^ | 3.88±0.14^bc^ | 2.41±0.02^d^ | 0.74±0.03^c^ |
|  | UAC | 200 W, RT, 60 min | 17.06±0.11^b^ | 1.24±0.09^a^ | 5.34±0.01^a^ | 3.48±1.42^a^ | 0.21±0.04^a^ | -0.04±0.04^c^ | 1.80±0.06^c^ | 4.69±0.22^a^ | 3.16±0.01^b^ | 0.83±0.02^b^ |

HB: Hot boiled, PO: Pour-over, 4CB: 4°C cold brewed coffee, 10CB:10 °C cold brewed coffee, UAC: Ultrasound-assisted cold brewed coffee, EY: Extraction yield, TDS: Total dissolved solids, TPC: Total phenolic extraction rate, TL: Total lipid extraction rate, Tpro: Total protein extraction rate, TA: Total acid extraction rate. Different letters indicate statistically significant differences (*P*<0.05) among treatments.

**Table S2.** Quantitative calibration curves of caffeine, chlorogenic acid, and trigonelline standards.

| **Compounds** | **Linear regression equations** | **Linear range (μg/mL)** | ***R^2^*** | **LOD**  **(μg/mL)** | **LOQ**  **(μg/mL)** |
| --- | --- | --- | --- | --- | --- |
| Caffeine | Y = 69046X - 7658 | 10-30 | 0.9975 | 0.31 | 0.86 |
| Chlorogenic acid | Y = 46175X + 38709 | 2.5-12.5 | 0.9992 | 0.80 | 2.31 |
| Trigonelline | Y = 9260.7X + 31495 | 50-200 | 0.9987 | 0.27 | 0.78 |

**Table S3.** Orthogonal optimization results of UAC.

| **L_9_ (3^3^)** | **Coffee powder to water ratio** | **Extraction time (min)** | **Ultrasonic power (W)** | **Extraction yield (%)** |
| --- | --- | --- | --- | --- |
| 1 | 1:12 | 20 | 200 | 11.79±1.12 |
| 2 | 1:12 | 30 | 100 | 10.89±0.81 |
| 3 | 1:12 | 60 | 150 | 13.57±0.28 |
| 4 | 1:15 | 20 | 150 | 14.07±0.27 |
| 5 | 1:15 | 30 | 200 | 15.21±0.31 |
| 6 | 1:15 | 60 | 100 | 17.72±0.53 |
| 7 | 1:18 | 20 | 100 | 16.28±0.26 |
| 8 | 1:18 | 30 | 150 | 16.23±0.18 |
| 9 | 1:18 | 60 | 200 | 14.80±0.14 |
| K1 | 12.08 | 14.05 | 14.96 |  |
| K2 | 15.67 | 14.11 | 14.62 |  |
| K3 | 15.77 | 15.36 | 13.93 |  |
| R | 3.69 | 1.31 | 1.03 |  |
| **Priorities order** | Coffee to water ratio > Extraction time > Ultrasonic power | | | |
| **Optimal level** | 1:15 | 60 | 100 |  |
| **Optimal group** | A_2_B_3_C_1_ | | | |

Ki, the average of the sum of experimental results in the same column of i level; R, range, R=Ki,_max_-Ki,_min_

**Table S4**. Physicochemical characteristics of coffee optimized by UAC methods.

| **Items** | | **Values** |
| --- | --- | --- |
| **EY/ %** | | 17.72±0.53 |
| **TDS/ °Brix** | | 1.44±0.06 |
| **pH** | | 5.37±0.02 |
| **Color** | **L*** | 3.31±0.17 |
|  | **a*** | 0.28±0.02 |
|  | **b*** | 0.85±0.06 |
| **TPC/ %** | | 1.83±0.04 |
| **TL/ %** | | 4.71±0.13 |
| **Tpro/ %** | | 3.14±0.02 |
| **TA/ %** | | 0.84±0.03 |
| **Caffeine/ mg/mL** | | 0.73±0.00 |
| **CGA/ mg/mL** | | 1.06±0.01 |
| **Trigonelline/ mg/mL** | | 0.52±0.00 |

EY: Extraction yield, TDS: Total dissolved solids, TPC: Total phenolic extraction rate, TL: Total lipid extraction rate, Tpro: Total protein extraction rate, TA: Total acid extraction rate. CGA: Chlorogenic acid.


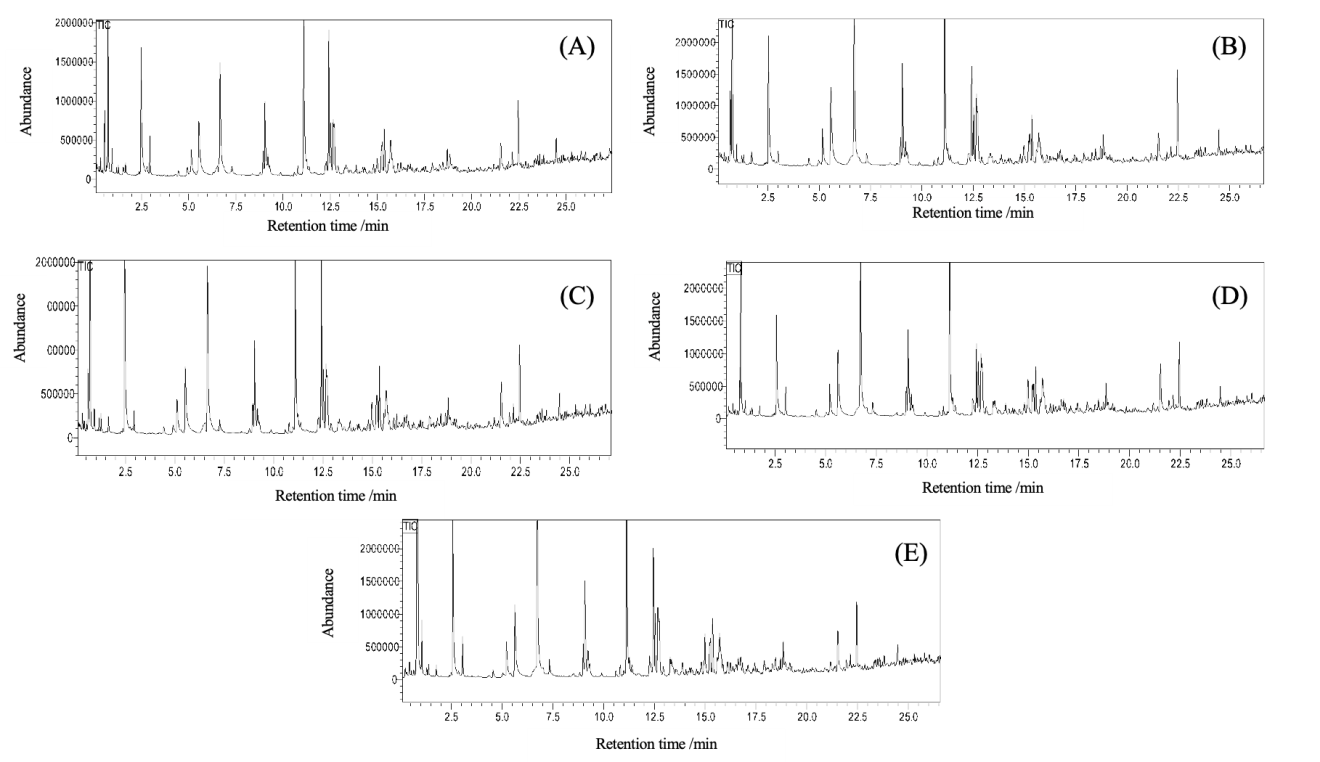


**Fig. S1**. Total ion chromatograms of volatile compounds in (A) hot boiled coffee extracts, (B) pour-over coffee extracts, (C) 4 °C cold brewed coffee extracts, (D) 10 °C cold brewed coffee extracts, and (E) ultrasound-assisted cold brewed coffee extracts.


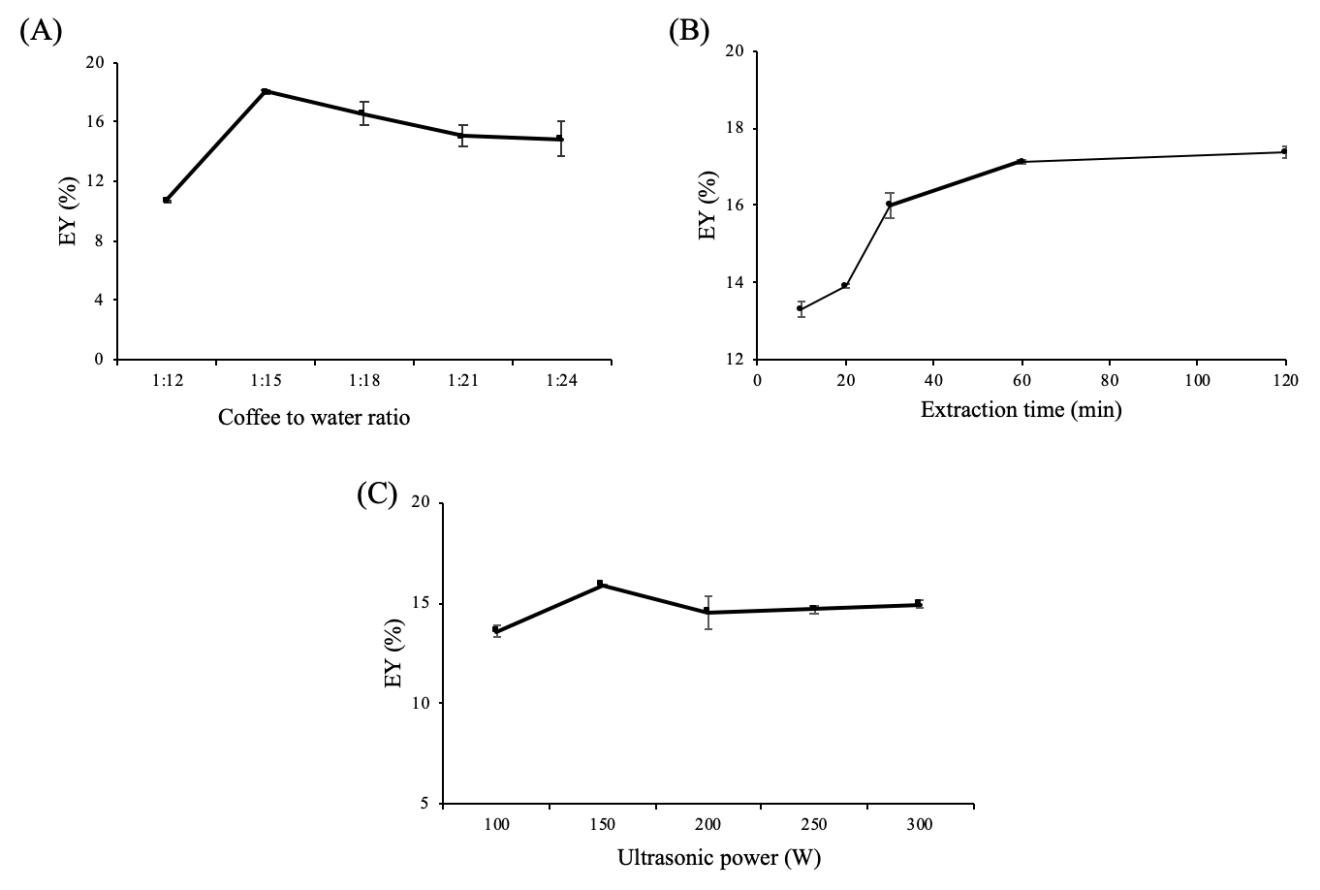


**Fig. S2**. The effect of (A) coffee to water ratio, (B) extraction time, and (C) ultrasonic power on coffee extraction yield (EY).
